# Rotavirus NSP1 Contributes to Intestinal Viral Replication, Pathogenesis, and Transmission

**DOI:** 10.1128/mBio.03208-21

**Published:** 2021-12-14

**Authors:** Gaopeng Hou, Qiru Zeng, Jelle Matthijnssens, Harry B. Greenberg, Siyuan Ding

**Affiliations:** a Department of Molecular Microbiology, Washington University School of Medicine, St. Louis, Missouri, USA; b KU Leuven-University of Leuven, Department of Microbiology, Immunology and Transplantation, Rega Institute for Medical Research, Leuven, Belgium; c VA Palo Alto Health Care System, Department of Veterans Affairs, Palo Alto, California, USA; d Department of Medicine, Division of Gastroenterology and Hepatology, Stanford School of Medicine, Stanford, California, USA; e Department of Microbiology and Immunology, Stanford School of Medicine, Stanford, California, USA; Virginia Polytechnic Institute and State University

**Keywords:** NSP1, double-stranded RNA virus, enteric viruses, innate immunity, interferons, reverse genetics, rotavirus

## Abstract

Rotavirus (RV)-encoded nonstructural protein 1 (NSP1), the product of gene segment 5, effectively antagonizes host interferon (IFN) signaling via multiple mechanisms. Recent studies with the newly established RV reverse genetics system indicate that NSP1 is not essential for the replication of the simian RV SA11 strain in cell culture. However, the role of NSP1 in RV infection *in vivo* remains poorly characterized due to the limited replication of heterologous simian RVs in the suckling mouse model. Here, we used an optimized reverse genetics system and successfully recovered recombinant murine RVs with or without NSP1 expression. While the NSP1-null virus replicated comparably with the parental murine RV in IFN-deficient and IFN-competent cell lines *in vitro*, it was highly attenuated in 5-day-old wild-type suckling pups in both the 129sv and C57BL/6 backgrounds. In the absence of NSP1 expression, murine RV had significantly reduced replication in the ileum, systemic spread to mesenteric lymph nodes, fecal shedding, diarrhea occurrence, and transmission to uninoculated littermates. The defective replication of the NSP1-null RV in small intestinal tissues occurred as early as 1 day postinfection. Of interest, the replication and pathogenesis defects of NSP1-null RV were only minimally rescued in *Stat1* knockout pups, suggesting that NSP1 facilitates RV replication in an IFN-independent manner. Our findings highlight a pivotal function of NSP1 during homologous RV infections *in vivo* and identify NSP1 as an ideal viral protein for targeted attenuation for future vaccine development.

## INTRODUCTION

Despite a dramatic reduction of rotavirus (RV)-associated morbidity and mortality following the introduction of multiple safe and effective RV vaccines, group A RVs remain a major cause of life-threatening gastroenteritis among young children from 1 month to 5 years old (1, 2). RV infections still result in approximately 128,500 to 215,000 deaths annually worldwide (3, 4). The RV vaccine option is also limited for immunocompromised children due to the risk of persistent shedding and diarrhea (5). Thus, there remains an urgent need to develop more effective vaccines, especially for immunosuppressed individuals.

Although RV infections in mammals occur frequently (6), RV isolates from one host species generally replicate less efficiently in heterologous species. Several RV-encoded factors, including VP3, VP4, NSP1, NSP2, and NSP3, have been implicated in contributing to this host range restriction phenotype (7). Among these viral proteins, NSP1 has been identified as an interferon (IFN) antagonist with several distinct mechanisms that enhances virus replication (8–12). NSP1 from many animal RV strains binds to and promotes the proteasomal degradation of interferon regulatory factor 3 (IRF3) (9, 13). NSP1 also recognizes and degrades IRF5, IRF7, and IRF9 (14, 15). NSP1 from several human and porcine RV strains binds to the host cullin-3 E3 ligase complex (16) and induces β-transduction repeat-containing protein (β-TrCP) degradation (17). In addition, NSP1 can directly target signal transducer and activator of transcription 1 (STAT1) phosphorylation and/or translocation into the nucleus to further block the IFN amplification pathway (10, 18). Taken together, all of these studies have established NSP1 as a potent inhibitor of the host IFN responses to facilitate RV replication.

With the development of a new plasmid-based RV reverse genetics (RG) system, several groups successfully rescued recombinant simian RVs (SA11 strain), including one in which NSP1 is almost completely replaced by a NanoLuc luciferase reporter except for the first 37 amino acids at the N terminus (19, 20). The replication of this recombinant SA11 is only modestly lower than the parental SA11 strain in MA104 cells, suggesting that NSP1 is dispensable for RV infection *in vitro*. However, since heterologous RVs replicate and spread inefficiently in mice (21), the role of NSP1 in RV infection under physiologically relevant conditions cannot be studied using this system. To overcome this hurdle, we recently constructed and recovered a fully replication-competent, infectious, and virulent recombinant murine RV using an optimized RG system (22). In this study, we take advantage of this modified RG system and further generate a new NSP1-deficient murine RV to directly address the significance and functional relevance of the NSP1 protein in intestinal replication and pathogenesis *in vivo*.

## RESULTS

### A recombinant NSP1-deficient murine RV can be successfully rescued via an optimized RG system.

**FIG 1:**
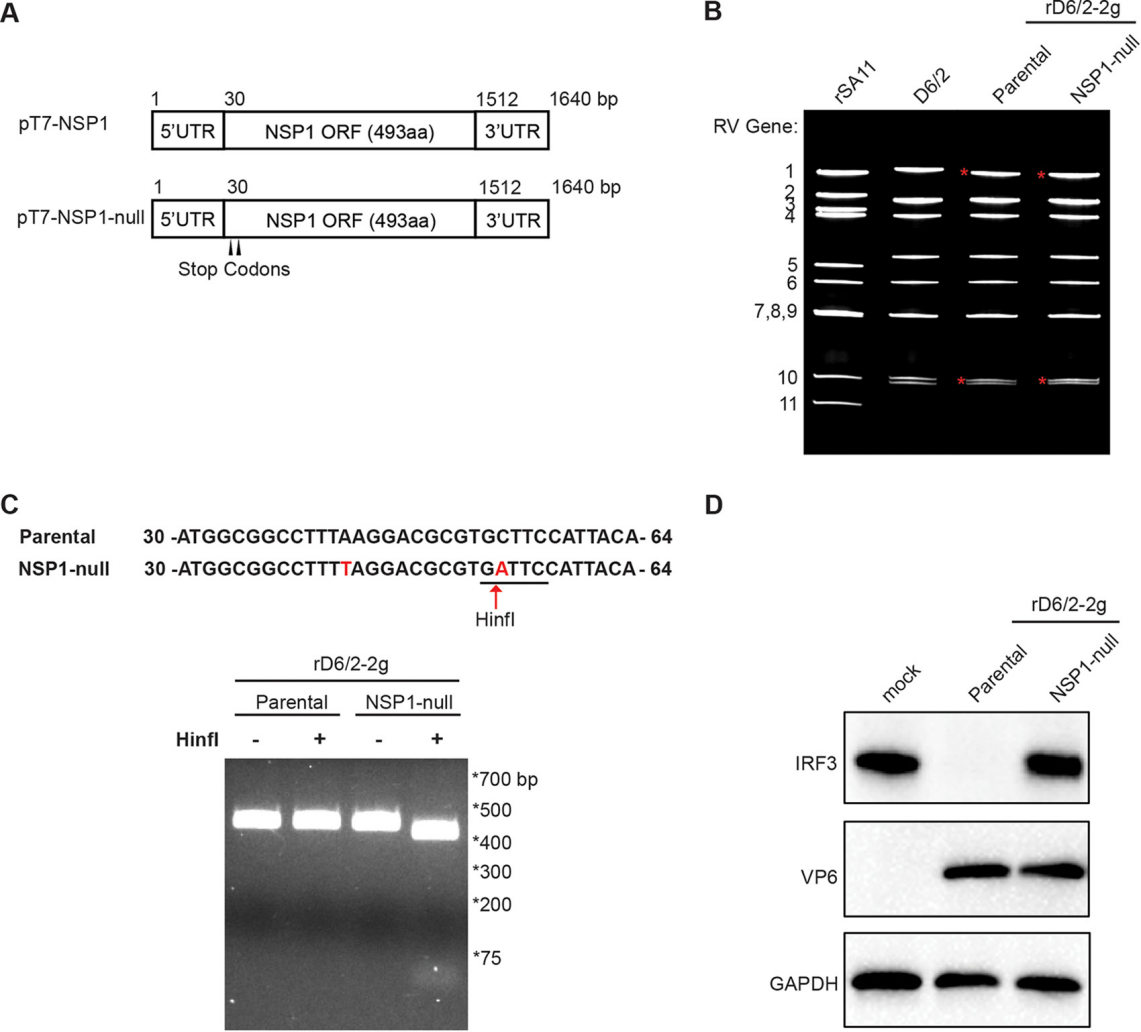
Generation of a recombinant NSP1-deficient murine RV using the optimized reverse genetics system. (A) Schematics of the plasmids used to rescue rD6/2-2g (pT7-NSP1) and NSP1-deficient rD6/2-2g-NSP1-null (pT7-NSP1-null) viruses. To generate the rD6/2-2g-NSP1-null virus, AAG and TGC at the nucleotide positions 43 to 45 and 52 to 55 were replaced with two stop codons, TAG and TGA, which are indicated by the black arrowheads. UTR, untranslated region; ORF, open reading frame; aa, amino acids. (B) RNA was extracted from sucrose gradient-concentrated indicated RV strains, separated on a 4 to 15% polyacrylamide gel, and stained by ethidium bromide. Genes 1 and 10 from the SA11 strain are marked by red asterisks. (C) The stop codon introduced at nucleotide positions 52 to 55 creates a unique HinfI digestion site. The NSP1 5′-end PCR products were digested by HinfI at 37°C for 1 h and separated by a 2% agarose gel. (D) MA104 cells were infected by parental rD6/2-2g or rD6/2-2g-NSP1-null at an MOI of 3 for 6 h. The infected cells were lysed by RIPA buffer, and the protein levels of IRF3, VP6, and GAPDH in the cell lysates were analyzed by immunoblotting using indicated antibodies.

To determine the role of NSP1 in viral replication *in vivo*, we utilized the recombinant D6/2 murine-like RV backbone with 2 gene segments (1 and 10) derived from the simian RV SA11 strain and the other 9 gene segments (including NSP1) from the murine RV D6/2 strain (designated here rD6/2-2g) (22). In order to generate an NSP1-deficient rD6/2-2g virus (rD6/2-2g-NSP1-null), we introduced two premature stop codons in gene segment 5 by replacing AAG and TGC at the nucleotide positions 43 to 45 and 52 to 55 with TAG and TGA, respectively, via site-directed mutagenesis (Fig. 1A). With these manipulations, the protein product of gene 5 from the rD6/2-2g-NSP1-null infection is limited to the first 4 amino acids. Using the optimized RG system, we succeeded in recovering a replication-competent rD6/2-2g-NSP1-null. We next extracted the viral RNA from sucrose cushion-purified RVs and performed polyacrylamide gel electrophoresis (PAGE) to analyze the viral genomic double-stranded RNA (dsRNA) segments. The genomic dsRNA migration patterns were identical between rD6/2-2g and rD6/2-2g-NSP1-null viruses, with the genes 1 and 10 from SA11 and the remaining 9 genes from D6/2 (Fig. 1B). We also validated the NSP1-null virus by a unique enzymatic digestion site (HinfI) introduced by the second stop codon. In comparison, cDNA amplified from gene segment 5 of rD6/2-2g was resistant to HinfI digestion (Fig. 1C). Lastly, we examined IRF3 degradation as a functional readout of murine RV NSP1 expression. To that end, we performed immunoblotting analysis to examine the cell lysates of MA104 cells infected by rD6/2-2g and rD6/2-2g-NSP1-null. With similar protein levels of RV VP6, indicating comparable replication between rD6/2-2g and rD6/2-2g-NSP1-null, the protein levels of IRF3 were undetectable in rD6/2-2g-infected MA104 cells whereas IRF3 was not degraded by rD6/2-2g-NSP1-null infection (Fig. 1D). Based on these results, we demonstrate that the rD6/2-2g-NSP1-null virus was successfully rescued and did not express the NSP1 protein.

### The replication of a recombinant NSP1-deficient murine RV is comparable to the parental murine RV in multiple cell lines.

**FIG 2:**
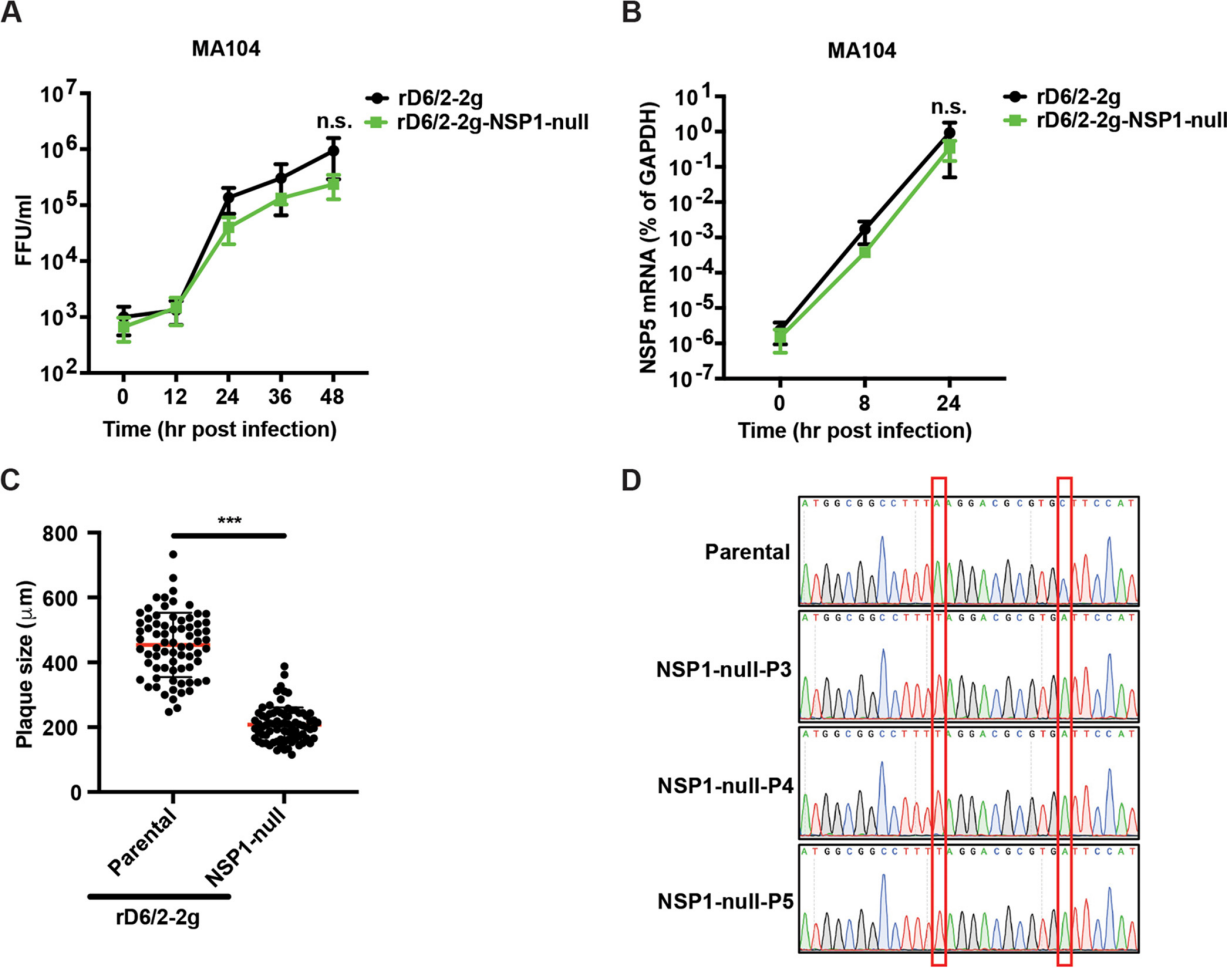
Replication kinetics of rD6/2-2g and rD6/2-2g-NSP1-null in MA104 cells. (A) MA104 cells were infected with rD6/2-2g or rD6/2-2g-NSP1-null at an MOI of 0.01 (FFU/cell) for 0, 12, 24, 36, and 48 h. Infected cells were harvested by freezing and thawing 3 times. The titers of the propagated viruses at different time points were determined by an FFU assay. The data displayed are the mean ± SD for three different assays. (B) MA104 cells were infected as described above for 0, 8, and 24 h. RNA was extracted from infected cells, and RT-qPCR was used to measure RV NSP5 transcript levels in infected cells. The data shown are the mean ± SD for three different assays. (C) Plaque assays were performed for rD6/2-2g or rD6/2-2g-NSP1-null in MA104 cells at an MOI of 0.01, and individual plaque formation was recorded and measured at 5 dpi by a bright-field microscope. The data shown are the mean ± SD for two different assays. (D) Indicated recombinant murine RVs were serially passaged 5 times in MA104 cells. The NSP1 fragments were amplified from the viruses and analyzed by Sanger sequencing. ***, *P* < 0.001; n.s., not significant (unpaired Student’s *t* test).

To determine whether the loss of NSP1 protein negatively impacts virus replication in cell culture, we performed a multistep growth curve for rD6/2-2g and rD6/2-2g-NSP1-null in MA104 cells at a multiplicity of infection (MOI) of 0.01. Both focus-forming unit (FFU) and quantitative reverse transcription-PCR (RT-qPCR) assays did not reveal significant differences between rD6/2-2g and rD6/2-2g-NSP1-null over the time course (Fig. 2A and B). The plaque sizes of rD6/2-2g-NSP1-null (diameter, 2.07 ± 0.53 mm) were significantly smaller than those of rD6/2-2g (diameter, 4.53 ± 0.99 mm) in MA104 cells (Fig. 2C). We also validated the genetic stability of rD6/2-2g-NSP1-null by serially passaging the virus 5 times in MA104 cells and confirming the presence of two stop codons in gene segment 5 (Fig. 2D). These results are consistent with the previous report that an intact NSP1 is not required for simian RV SA11 strain infection in MA104 cells (19, 20). To test whether the lack of NSP1 results in a minor replication defect that was not apparent in cell culture, we carried out competition experiments in MA104 cells coinfected with the same titers of rD6/2-2g and rD6/2-2g-NSP1-null viruses. At either low MOI (0.01) or high MOI (3), rD6/2-2g did not outgrow rD6/2-2g-NSP1-null RV at 8 and 24 h postinfection (hpi), suggesting that the replication ability of rD6/2-2g-NSP1-null was comparable to rD6/2-2g (see Fig. S1 in the supplemental material).

10.1128/mBio.03208-21.1 FIG S1 Sanger sequencing chromatograms of PCR products for the competition assay of rD6/2-2g and rD6/2-2g-NSP1-null. MA104 cells were infected by rD6/2-2g and rD6/2-2g-NSP1-null at an MOI of 0.01 for 1, 8, and 24 h or at an MOI of 3 for 1 and 8 h. Total RNA was extracted, and amplified NSP1 fragments were analyzed using Sanger sequencing. Download FIG S1, TIF file, 1.3 MB. Copyright © 2021 Hou et al. 2021 Hou et al. https://creativecommons.org/licenses/by/4.0/ This content is distributed under the terms of the Creative Commons Attribution 4.0 International license.

**FIG 3:**
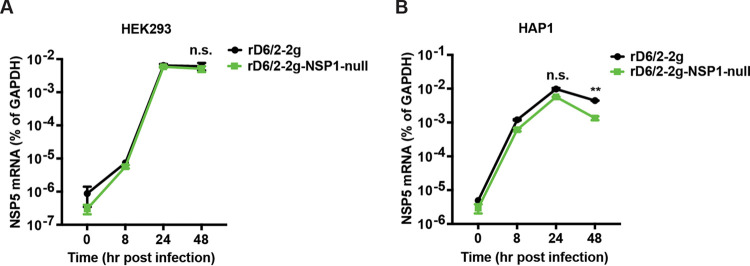
Growth curves of rD6/2-2g and rD6/2-2g-NSP1-null in IFN-competent cells. HEK293 (A) and HAP1 (B) cells were infected by rD6/2-2g and rD6/2-2g-NSP1-null at an MOI of 0.01 for 0, 8, 24, and 48 h. The expression level of NSP5 was quantified by RT-qPCR. The data shown are the mean ± SD for three individual assays. **, *P* < 0.01; n.s., not significant (unpaired Student’s *t* test).

Since NSP1 dampens the host IFN responses, which are defective in MA104 cells, we next tested whether the replication of rD6/2-2g-NSP1-null is restricted in IFN-competent cell lines. We examined the growth kinetics of rD6/2-2g and rD6/2-2g-NSP1-null in two different cell types: HEK293 and HAP1 cells, which are a human embryonic fibroblastic cell line and a human myeloid leukemia cell line, respectively. Both cell types are capable of mounting robust type I and III IFN responses to RV infection (23). However, the rD6/2-2g-NSP1-null mutant still replicated comparably to rD6/2-2g in these IFN-competent cell lines (Fig. 3A and B), suggesting that NSP1 is dispensable for murine RV replication in an IFN competency-independent manner.

### Loss of NSP1 severely attenuates murine RV replication in 129sv suckling mice.

**FIG 4:**
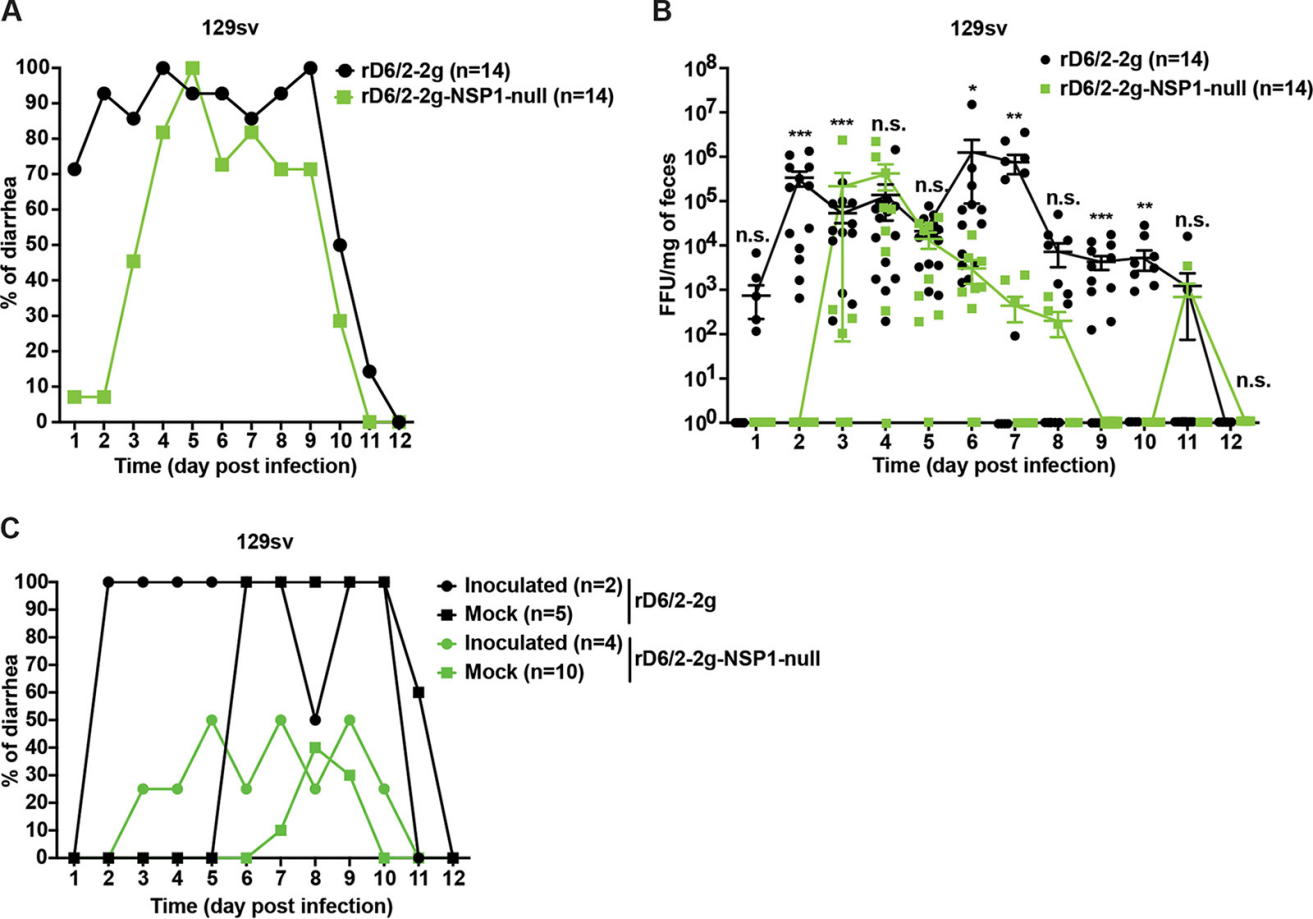
Characterization of diarrhea, fecal shedding, and transmission of rD6/2-2g and rD6/2-2g-NSP1-null in wild-type 129sv mice. (A) Five-day-old 129sv mice were orally inoculated with 1.5 × 10^3^ FFU of rD6/2-2g and rD6/2-2g-NSP1-null. Diarrheal development was recorded from days 1 to 12 postinfection. “n” indicates the number of mice used in each group. (B) Fecal shedding of the infectious RVs was monitored by an FFU assay and normalized by the weight of feces. Virus shedding within the same group on each day is shown as mean ± SEM. (C) To evaluate the transmission ability of rD6/2-2g, 2 pups were orally infected with 1.5 × 10^3^ FFU of rD6/2-2g, and 5 uninfected suckling littermates were cohoused with the inoculated pups in the same cage. For rD6/2-2g-NSP1-null, 2 pups in one cage and 2 in another cage were orally infected with the rD6/2-2g-NSP1-null virus described above and cohoused with 6 and 4 uninoculated pups, respectively. Diarrhea was evaluated until 12 dpi as described above. *, *P* < 0.05; **, *P* < 0.01; ***, *P* < 0.001; n.s., not significant (two-way ANOVA).

To compare the replication, pathogenesis, and spread of rD6/2-2g and rD6/2-2g-NSP1-null in an *in vivo* environment, we orally inoculated 5-day-old wild-type 129sv suckling mice with 1.5 × 10^3^ FFU of rD6/2-2g or rD6/2-2g-NSP1-null and monitored the diarrheal development from day 1 to 12 postinfection. While the diarrhea occurrence from the rD6/2-2g-infected group was consistently higher than 70% for the first 10 days, rD6/2-2g-NSP1-null caused minimal to no diarrhea in infected animals for the first 2 days (Fig. 4A). Starting from 3 days postinfection (dpi), rD6/2-2g-NSP1-null started to approximate the parental virus, with their curves eventually trending in a similar fashion (Fig. 4A). We also quantified the shedding of infectious RVs in the feces of mouse pups by an FFU assay. Consistent with the defects in causing diarrhea, fecal shedding of rD6/2-2g-NSP1-null in infected mice could not be detected at 1 and 2 dpi, whereas we observed high RV titers from the rD6/2-2g infection (Fig. 4B). The two virus shedding curves looked similar from 3 to 5 dpi, before the RV shedding in rD6/2-2g-NSP1-null-infected pups waned again compared to that of the rD6/2-2g group (Fig. 4B). Furthermore, we also evaluated the ability of these two viruses to transmit to uninfected littermates, an important trait for viruses that spread fecal-orally. We found that virtually all the noninoculated littermates of the rD6/2-2g-infected pups developed diarrhea at 6 dpi (Fig. 4C). In comparison, the maximal percentage of diarrhea among mock-infected pups in the rD6/2-2g-NSP1-null cage reached 40% and lasted only 1 day (Fig. 4C). Collectively, these results suggest that NSP1 is necessary for optimal RV infection, disease, and spread *in vivo*.

**FIG 5:**
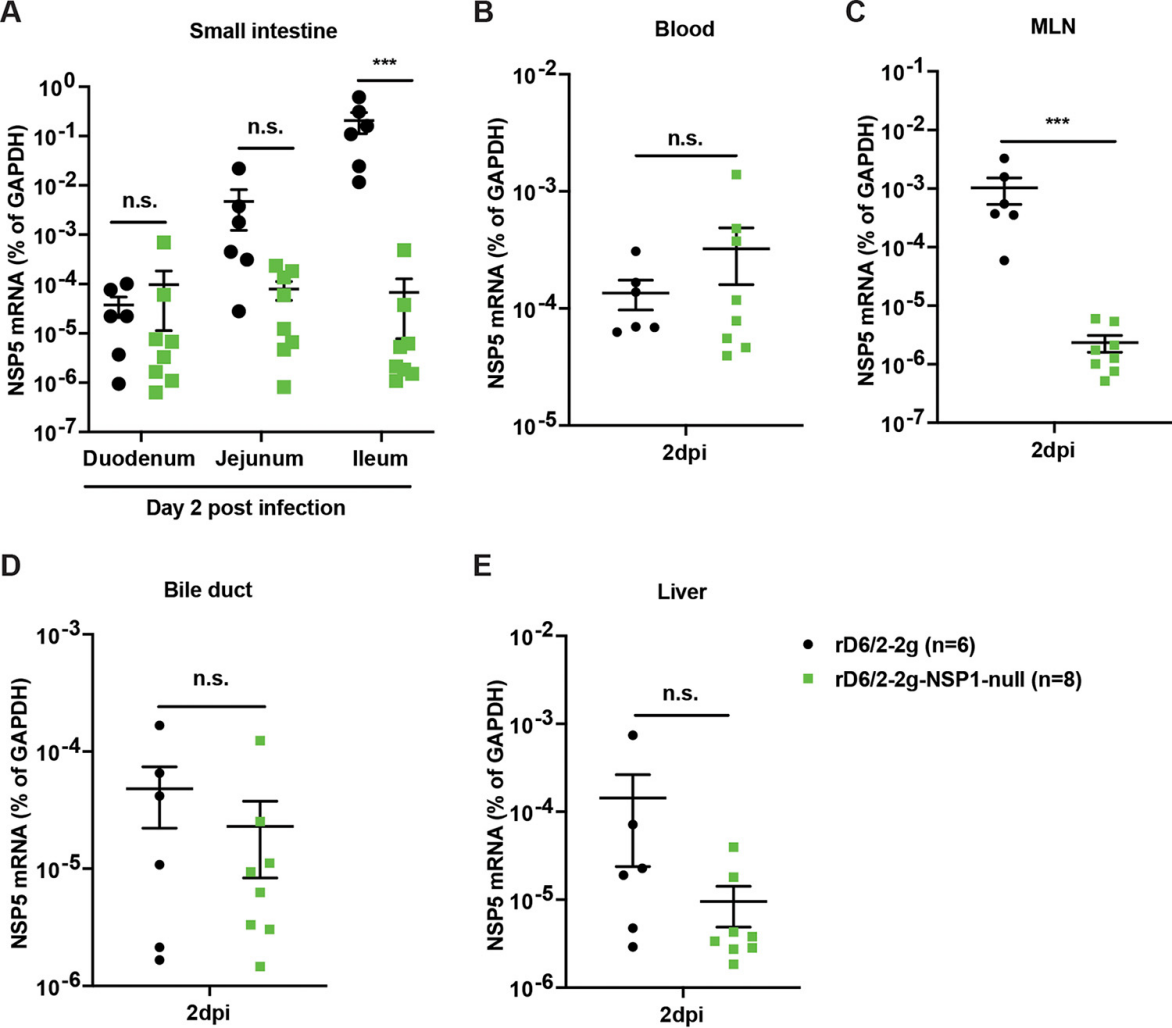
Viral loads of rD6/2-2g and rD6/2-2g-NSP1-null in the small intestines and indicated systemic sites in wild-type 129sv mice. (A) Five-day-old wild-type 129sv pups were orally infected with 1.5 × 10^3^ FFU of rD6/2-2g and rD6/2-2g-NSP1-null. RNA was extracted from duodenum, jejunum, and ileum collected at 2 dpi, and RT-qPCR was used to detect RV NSP5 mRNA levels. (B to E) Same as panel A except that blood, MLNs, bile duct, and liver were collected instead. ***, *P* < 0.001; n.s., not significant (Mann-Whitney U test for panel A and unpaired Student’s *t* test for panels B to E).

To directly investigate whether NSP1 contributes to RV intestinal replication, we collected all three small intestinal segments (i.e., duodenum, jejunum, and ileum) from rD6/2-2g- and rD6/2-2g-NSP1-null-infected pups at 2 dpi and measured viral loads by RT-qPCR. The number of rD6/2-2g genome copies in the ileum was significantly (about 3 logs) higher than that of rD6/2-2g-NSP1-null, despite no major differences in the duodenum and jejunum (Fig. 5A). We also found that the viral loads of rD6/2-2g in mesenteric lymph nodes (MLNs) were higher than those of rD6/2-2g-NSP1-null, whereas no difference were observed in the blood, bile ducts, or liver (Fig. 5B to E).

**FIG 6:**
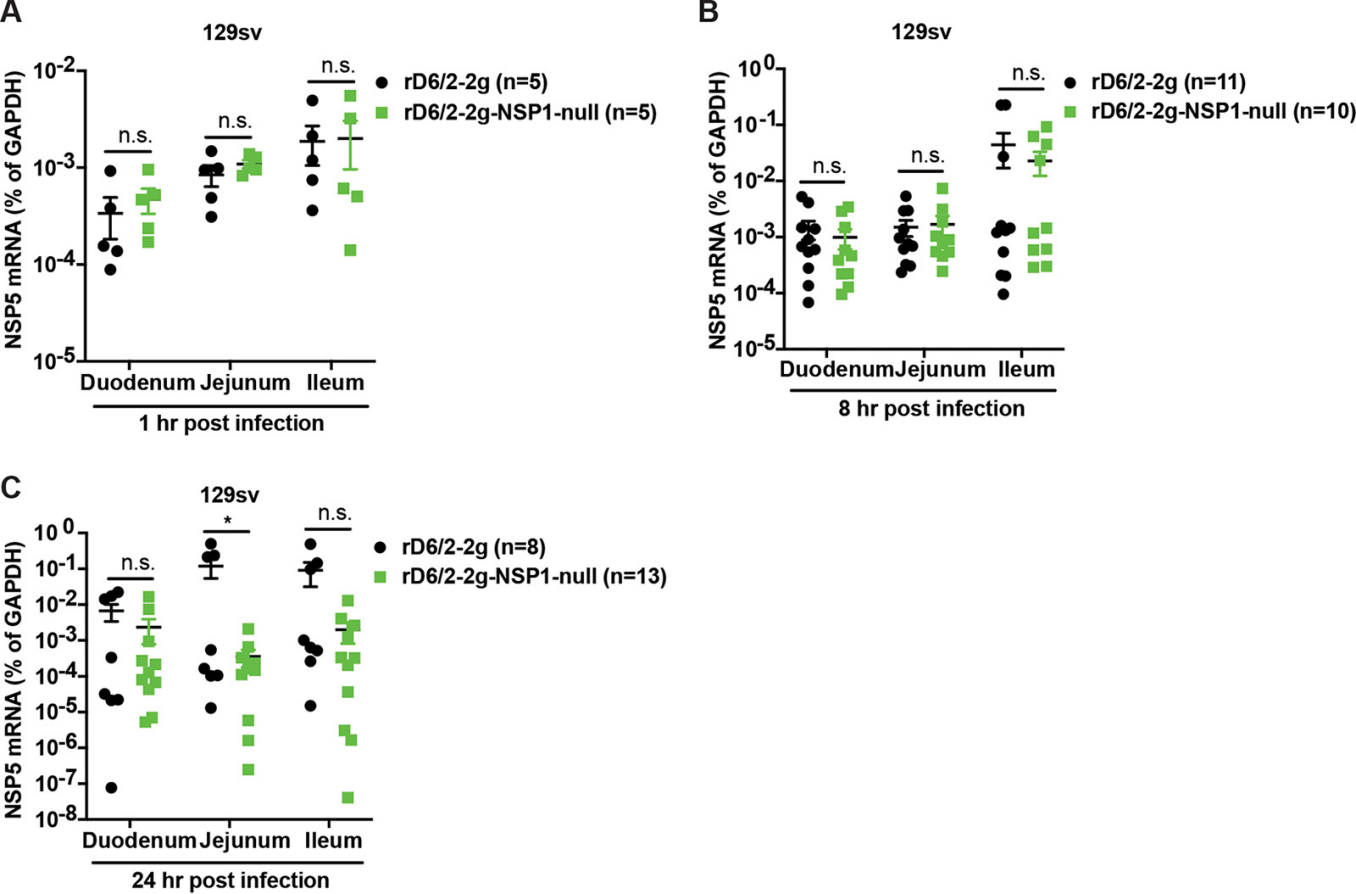
Viral loads of rD6/2-2g and rD6/2-2g-NSP1-null in the small intestines at different time points in wild-type 129sv mice. (A) Five-day-old wild-type 129sv pups were orally infected with 1.5 × 10^3^ FFU of rD6/2-2g and rD6/2-2g-NSP1-null. RNA was extracted from duodenum, jejunum, and ileum collected at 1 h postinoculation, and RT-qPCR was used to detect RV NSP5 mRNA levels. (B and C) Same as panel A except that duodenum, jejunum, and ileum were collected at 8 and 24 h postinoculation instead. *, *P* < 0.05; n.s., not significant (Mann-Whitney U test).

We next performed an *in vivo* time course experiment to further characterize the attenuated intestinal replication of rD6/2-2g-NSP1-null. With similar input virus titers throughout the small intestine at 1 hpi, the replication levels of rD6/2-2g and rD6/2-2g-NSP1-null were comparable at 8 hpi (approximately one replication cycle) as measured by RT-qPCR (Fig. 6A and B). In contrast, rD6/2-2g genome copies in the jejunum were significantly (more than 2 logs) higher than those of rD6/2-2g-NSP1-null at 24 hpi (Fig. 6C). Although not statistically significant, the viral load of rD6/2-2g in the ileum was also more than 10-fold higher than that of rD6/2-2g-NSP1-null, suggesting that NSP1 was required for murine RV infection to successfully propagate *in vivo* by 24 hpi.

### Revertant NSP1 mutations rescued NSP1-deficient murine RV replication in mice.

Notably, we noticed that 4 out of 14 mice from the rD6/2-2g-NSP1-null-infected group shed large amounts of infectious viruses in the feces at 3 dpi (Fig. 4B). This was reproducibly observed in two independent sets of experiments (6 out of 7 at 4 dpi in one experiment and 4 out of 7 at 3 dpi in the other experiment) (Fig. S2). To account for this enhanced replication, we amplified the 5′ end of gene 5 (17 to 466 nucleotides) directly from the stool samples collected from mice that had detectable RV shedding from 1 to 4 dpi and performed Sanger sequencing. While the rD6/2-2g-NSP1-null RV stock used for inoculation carried the mutations (Fig. S3A), NSP1 fragments amplified from the feces of the rD6/2-2g-NSP1-null-infected mice were indistinguishable from those from rD6/2-2g infection (Fig. S3B), indicating that RV in the fecal specimens readily reverted to the wild-type sequences. While we could not detect any RV shedding from rD6/2-2g-NSP1-null-infected mice at 1 or 2 dpi, 75% of mice that shed infectious RVs at 3 dpi had complete NSP1 reversion mutations and 25% had incomplete reversion (Fig. S3B). Six out of 14 mice infected with rD6/2-2g-NSP1-null shed virus at 4 dpi, and all 6 animals had wild-type NSP1 sequences (Fig. S3B). These data are consistent with our observation that the rD6/2-2g-NSP1-null-infected mice unexpectedly developed diarrhea starting from 3 dpi and further emphasize an indispensable role that NSP1 protein plays during virus replication *in vivo*.

10.1128/mBio.03208-21.2 FIG S2 Characterization of the virus shedding of rD6/2-2g and rD6/2-2g-NSP1-null in wild-type 129sv mice. (A) Five-day-old 129sv mice were orally inoculated with 1.5 × 10^3^ FFU of rD6/2-2g and rD6/2-2g-NSP1-null. Viral shedding in stool samples were detected by an FFU assay and normalized by the feces weight. (B) Another two cages of suckling mice were orally infected as in panel A, and the fecal shedding was measured and normalized as described above. Virus shedding within the same group on each day is shown as mean ± SEM. ***, *P* < 0.001 (two-way ANOVA). Download FIG S2, TIF file, 0.1 MB. Copyright © 2021 Hou et al. 2021 Hou et al. https://creativecommons.org/licenses/by/4.0/ This content is distributed under the terms of the Creative Commons Attribution 4.0 International license.

10.1128/mBio.03208-21.3 FIG S3 Sanger sequencing of the RV gene 5 fragments and pie chart summary of the NSP1 reversion. (A) NSP1 fragments (17 to 466 nucleotides) were amplified from the rD6/2-2g and rD6/2-2g-NSP1-null RV stocks and analyzed by Sanger sequencing. (B) Same as panel A except that feces from rD6/2-2g-NSP1-null-inoculated wild-type 129sv mice at days 3 and 4 postinfection were analyzed and plotted as percentage of reversion. (C) Same as panel A except that feces from rD6/2-2g-NSP1-null-inoculated *Stat1* KO 129sv mice at days 1 to 4 postinfection were analyzed instead. Download FIG S3, TIF file, 0.3 MB. Copyright © 2021 Hou et al. 2021 Hou et al. https://creativecommons.org/licenses/by/4.0/ This content is distributed under the terms of the Creative Commons Attribution 4.0 International license.

### The blunted replication and pathogenesis of the recombinant NSP1-deficient murine RV are only partially recovered in the *Stat1* KO mice.

**FIG 7:**
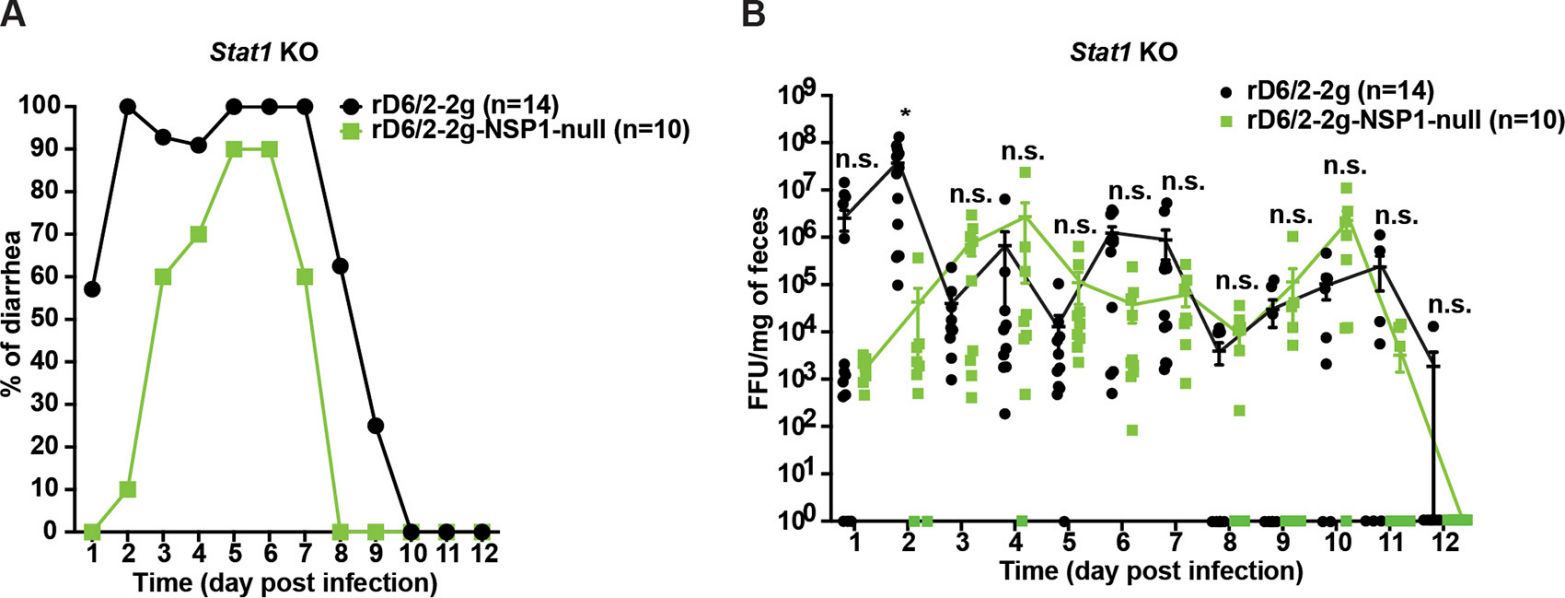
Characterization of diarrhea and fecal shedding of rD6/2-2g and rD6/2-2g-NSP1-null in *Stat1* KO 129sv mice. (A) Five-day-old *Stat1* KO 129sv mice were orally inoculated with 1.5 × 10^3^ FFU of rD6/2-2g and rD6/2-2g-NSP1-null. The diarrhea rate was monitored from days 1 to 12 postinfection. (B) Viral shedding in stool samples was detected by an FFU assay and normalized by the feces weight. Virus shedding within one group on each day is shown as mean ± SEM. *, *P* < 0.05; n.s., not significant (two-way ANOVA).

Since NSP1 functions as a highly potent IFN antagonist *in vitro*, we reasoned that rD6/2-2g-NSP1-null may be attenuated in wild-type 129sv mice due to the lack of IFN-inhibitory capacity. To test this hypothesis, we orally infected 5-day-old *Stat1* knockout (KO) 129sv suckling pups, unable to respond to type I, II, and III IFNs, with 1.5 × 10^3^ FFU of rD6/2-2g or rD6/2-2g-NSP1-null. For both viruses, the overall diarrheal development patterns in infected *Stat1* KO 129sv mice resembled those in wild-type mice (Fig. 7A). However, compared to rD6/2-2g-infected animals that developed 57% and 100% diarrhea at 1 and 2 dpi, respectively, there was little to no diarrhea from rD6/2-2g-NSP1-null-inoculated animals (Fig. 7A). We also evaluated the fecal RV shedding in the infected mice by an FFU assay. Even in the absence of IFN signaling, at 1 and 2 dpi, RV shedding of rD6/2-2g remained >3 logs higher than that from the rD6/2-2g-NSP1-null infection (Fig. 7B), suggesting that NSP1 may facilitate RV replication in an IFN-independent manner.

**FIG 8:**
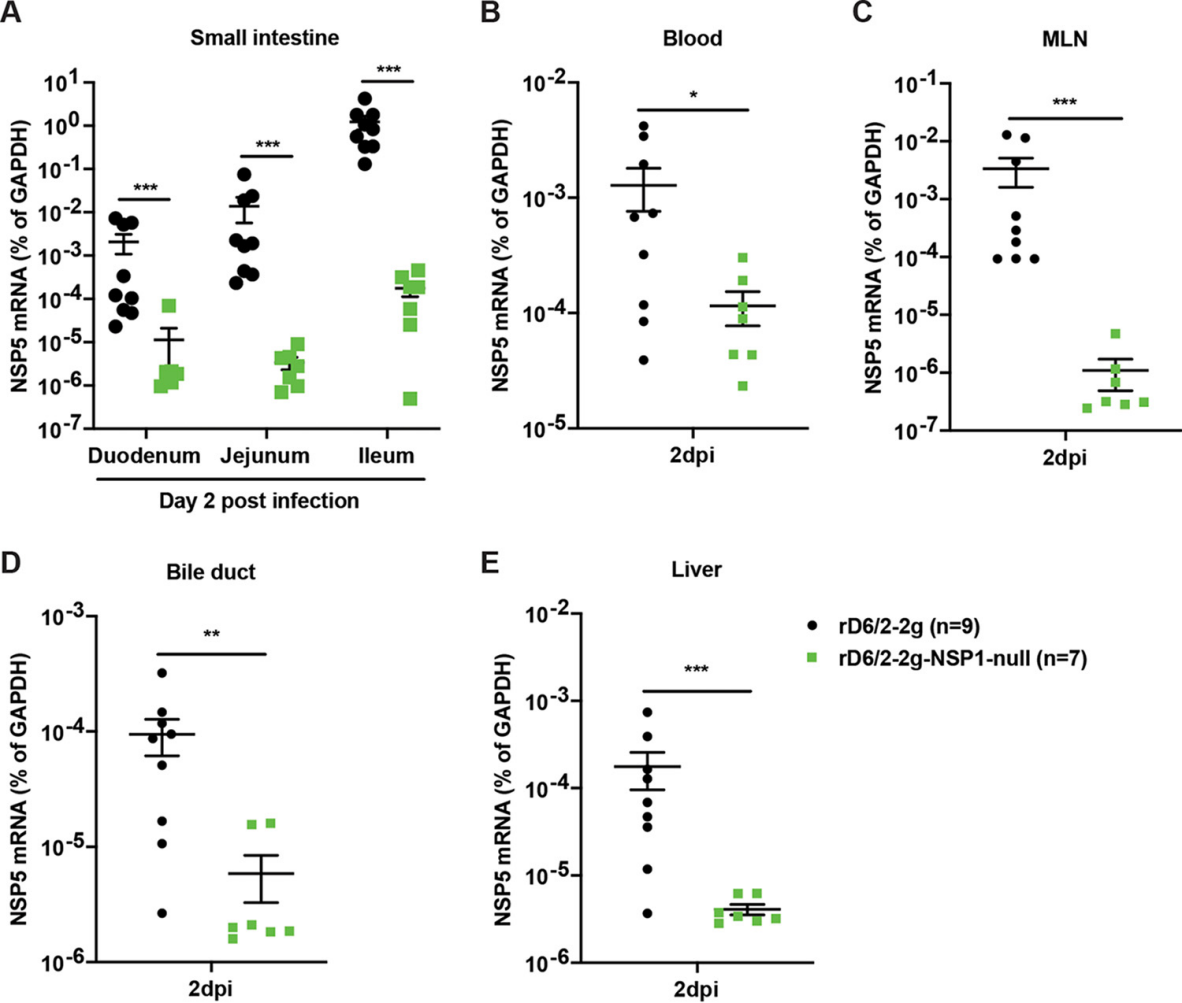
Viral loads of rD6/2-2g and rD6/2-2g-NSP1-null in the small intestines and indicated extraintestinal tissues in *Stat1* KO 129sv mice. (A) Five-day-old *Stat1* KO 129sv pups were orally infected with 1.5 × 10^3^ FFU of rD6/2-2g and rD6/2-2g-NSP1-null. The duodenum, jejunum, and ileum were collected at 2 dpi, and RV NSP5 mRNA levels were detected by RT-qPCR. (B to E) Same as panel A except that blood, MLNs, bile duct, and liver were collected instead. *, *P* < 0.05; **, *P* < 0.01; ***, *P* < 0.001; n.s., not significant (Mann-Whitney U test for panel A and unpaired Student’s *t* test for panels B to E).

Consistent with the diarrhea and fecal shedding results, we found that for all the small intestinal tissues examined, rD6/2-2g-NSP1-null was still severely attenuated compared to rD6/2-2g in *Stat1* KO mice at 2 dpi (Fig. 8A). The viral loads of rD6/2-2g in the ileum were approximately 7,000-fold higher than those of rD6/2-2g-NSP1-null-infected mice (Fig. 8A). Furthermore, rD6/2-2g also had significantly more spread to systemic organs including the blood, MLNs, bile duct, and liver than rD6/2-2g-NSP1-null (Fig. 8B to E). Collectively, these data indicate that even in a host devoid of IFN signaling, the replication of a murine RV without NSP1 expression remains highly attenuated *in vivo*.

Further supporting an IFN-independent role of NSP1 is the fact that rD6/2-2g-NSP1-null also reverted to the wild-type sequences in *Stat1* KO mice. We surveyed over 30 fecal samples from the rD6/2-2g-NSP1-null-infected mice. Out of the 8 mice that shed infectious RVs at 1 dpi, 25% of the mice had complete NSP1 revertant mutations and 75% of the mice had incomplete reversion (Fig. S3C). At 4 dpi, 8 out of the 10 mice that had substantial fecal shedding had viruses with wild-type NSP1 sequences (Fig. S3C). Therefore, all NSP1-deficient viruses reached 100% reversion in *Stat1* KO 129sv mice by 4 dpi, the same as that in the wild-type 129sv mice.

### The attenuated replication of the recombinant NSP1-deficient murine RV is not rescued in the *Asc* knockout mice.

Since inflammasome signaling inhibits RV infection and RV viral loads are significantly enhanced in the small intestines of *Asc* KO mice (24), which lack a key inflammasome adaptor protein, we next tested whether NSP1 acts to block inflammasome signaling. We orally infected 5-day-old wild-type C57BL/6 and *Asc* KO suckling pups with rD6/2-2g or rD6/2-2g-NSP1-null with 1.5 × 10^3^ FFU and harvested the small intestines at 24 hpi.

Historically, we have noticed that C57BL/6 mice develop less severe diarrhea than 129sv pups of the same age (25). Consistent with these findings, we observed lower levels of rD6/2-2g replication in the small intestines of C57BL/6 pups compared to 129sv pups (Fig. S4A). Importantly, the viral loads of rD6/2-2g in the jejunum were significantly higher than those of rD6/2-2g-NSP1-null-inoculated wild-type C57BL/6 mice (Fig. S4A), suggesting that a key function of NSP1 is required, irrespective of the mouse genetic background. Further, in *Asc* KO C57BL/6 mice, the viral loads of rD6/2-2g in the jejunum were still significantly higher than those of rD6/2-2g-NSP1-null-inoculated mice (Fig. S4B). These results indicate that the attenuated intestinal replication ability of NSP1-deficient RV may not be linked to host inflammasome signaling.

10.1128/mBio.03208-21.4 FIG S4 Viral loads of rD6/2-2g and rD6/2-2g-NSP1-null in the small intestines in wild-type and *Asc* KO C57BL/6 mice. (A) Five-day-old wild-type C57BL/6 pups were orally infected with 1.5 × 10^3^ FFU of rD6/2-2g and rD6/2-2g-NSP1-null RVs. The duodenum, jejunum, and ileum were collected at 24 hpi, and RV NSP5 mRNA levels were detected by RT-qPCR. (B) Same as panel A except that *Asc* KO C57BL/6 pups were used. *, *P* < 0.05; n.s., not significant (Mann-Whitney U test). Download FIG S4, TIF file, 0.09 MB. Copyright © 2021 Hou et al. 2021 Hou et al. https://creativecommons.org/licenses/by/4.0/ This content is distributed under the terms of the Creative Commons Attribution 4.0 International license.

### The attenuated replication of the recombinant NSP1-deficient simian RV SA11 is not rescued in *Stat1* KO mice.

To further corroborate the unexpected finding that NSP1 is required for RV replication in *Stat1* KO mice, we turned to the heterologous simian RV SA11 strain that leads to low but detectable fecal shedding when the mice are inoculated at a high dose (1 × 10^7^ PFU) (21). We successfully rescued an NSP1-deficient SA11 strain (rSA11-NSP1-null), which was given to 5-day-old wild-type and *Stat1* KO 129sv mice in direct comparison to the parental rSA11 at 1 × 10^7^ PFU. The overall trends of diarrheal development between rSA11 and rSA11-NSP1-null in wild-type 129sv mice were similar (Fig. S5A). In *Stat1* KO mice, both viruses developed less diarrhea than in the wild-type animals but the rSA11-NSP1-null seemed to be even more attenuated than rSA11 (Fig. S5A). We also quantified RV antigen shedding in the stool samples collected from rSA11- or rSA11-NSP1-null-infected mice by an enzyme-linked immunosorbent assay (ELISA). While neither virus had detectable shedding in wild-type pups, we observed that rSA11 but not rSA11-NSP1-null resulted in transient shedding between 4 and 6 dpi (Fig. S5B). Consistently, we found higher viral loads in the small intestines upon rSA11 infection than upon rSA11-NSP1-null infection of *Stat1* KO mice (Fig. S5C). Taken together, these data suggest that despite limited replication ability, the heterologous simian RV SA11 strain is also attenuated without NSP1 expression in IFN-deficient animals, resembling what we observed with homologous murine RV infections.

10.1128/mBio.03208-21.5 FIG S5 Characterization of diarrhea, fecal shedding, and replication of heterologous rSA11 and rSA11-NSP1-null in wild-type and *Stat1* KO 129sv mice. (A) Five-day-old wild-type and *Stat1* KO 129sv mice were orally infected with 1 × 10^7^ PFU of rSA11 and rSA11-NSP1-null. Diarrheal development was recorded from days 0 to 11 postinoculation. (B) Stool samples were collected from 1 to 12 days postinfection, and virus shedding in feces was measured by ELISA and plotted as optical density (O.D.) values. *, *P* < 0.05; **, *P* < 0.01; ***, *P* < 0.001; n.s., not significant (two-way ANOVA). (C) Small intestinal tissues were collected at 5 dpi. Total RNA was extracted, and RV NSP5 mRNA levels were detected by RT-qPCR. n.s., not significant (unpaired Student’s *t* test). Download FIG S5, TIF file, 0.2 MB. Copyright © 2021 Hou et al. 2021 Hou et al. https://creativecommons.org/licenses/by/4.0/ This content is distributed under the terms of the Creative Commons Attribution 4.0 International license.

## DISCUSSION

In this study, we exploited a recently developed and further optimized plasmid-based RG system to examine the role of NSP1 in RV replication *in vivo*. Previous studies showed that naturally isolated RV SA11-5S and SA11-30-1A variants (26), and some RG-rescued recombinant RVs, albeit unable to induce IRF3 degradation, replicate efficiently in cell culture (20). However, because of the limited replication and transmission of nonmurine heterologous RVs in mice (27), until now it has been difficult to assess whether NSP1 is required for RV replication *in vivo*. Here, taking advantage of an optimized RG system, we rescued an NSP1-deficient murine virus-like rD6/2-2g-NSP1-null virus (Fig. 1), which replicated similarly to rD6/2-2g in MA104 cells (Fig. 2A and B). The plaques formed by rD6/2-2g-NSP1-null were, however, much smaller than those of rD6/2-2g (Fig. 2C), reminiscent of the data derived from the SA11 strain (19, 20). The smaller plaque size may reflect a defect in the efficiency of cell-cell spread.

The similar growth properties of rD6/2-2g-NSP1-null and rD6/2-2g in MA104 cells may be due to IFN-defective MA104 cells, which also supported comparable propagation of the parental rSA11 strain and recombinant rSA11-dC103 and rSA11-Nluc that both lack an intact NSP1 expression. The replication of rSA11-Nluc in IFN-competent cells was, however, much lower than that of rSA11 (19, 28), and this phenotype may be caused by the missing IRF3 degradation ability of rSA11-dC103 and rSA11-Nluc. Thus, in order to assess whether NSP1 was important to murine RV replication in IFN-competent cells, we examined the growth curves of rD6/2-2g-NSP1-null and rD6/2-2g in HEK293 and HAP1 cells. Intriguingly, these two viruses still exhibited similar replication properties (Fig. 3). Although we observed a 3- to 4-fold-higher titer of rD6/2-2g in HAP1 cells at 48 hpi, there were no statistically significant differences at any other time points (Fig. 3). These results raise the possibilities that another RV protein may compensate for the loss of NSP1 or that the IFN-mediated antiviral activities are both cell type and virus strain specific.

Recent studies have reported that the recombinant murine-like RV (rD6/2-2g) replicates robustly in the small intestines of 129sv suckling pups, that rD6/2-2g infection causes diarrheal diseases, and that the transmission efficiency of rD6/2-2g is similar to that of the original reassortant murine RV D6/2 strain (22). Here, we orally inoculated the 129sv mice with rD6/2-2g or rD6/2-2g-NSP1-null. The lower percentage of diarrheal disease and lower titer of RV shedding in rD6/2-2g-NSP1-null-infected pups at the early time points revealed that NSP1 protein is important for virus pathogenesis *in vivo*. However, starting on 3 dpi, the curves of diarrhea and virus shedding of rD6/2-2g-NSP1-null approached those of the rD6/2-2g parental strain. This observation led us to ask whether the rD6/2-2g-NSP1-null reverted to wild-type rD6/2-2g, despite the presence of two stop codons inserted at the very beginning of the NSP1 open reading frame. To our surprise, all of the gene 5 segments amplified from rD6/2-2g-NSP1-null-infected 129sv mice examined at 4 dpi had completely reverted to wild-type sequences (see Fig. S3B in the supplemental material). We performed the infection experiments with rD6/2-2g and rD6/2-2g-NSP1-null at different time points, and these data were repeated in two independent experiments (Fig. S2); thus, the reversion observed in rD6/2-2g-NSP1-null-inoculated mice is unlikely to be due to the contamination by rD6/2-2g. We also amplified NSP1 from fecal specimens obtained on days 1 to 4 postinfection from *Stat1* KO 129sv mice. Two out of 8 mice had complete NSP1 reversion mutations, and the remaining 6 had incomplete reversion as early as 1 dpi; meanwhile, all the 8 *Stat1* KO 129sv mice had complete NSP1 reversion mutations at 4 dpi (Fig. S3C). These results emphasized the essential role of NSP1 protein during RV replication *in vivo*. We found significantly higher levels of rD6/2-2g in the small intestines, blood, MLNs, bile duct, and liver compared with rD6/2-2g-NSP1-null-infected *Stat1* KO 129sv mice at 2 dpi (Fig. 8), although the only statistically significant differences examined in the wild-type 129sv mice at 2 dpi were in the ileum and MLNs (Fig. 5). We found that the replication of rD6/2-2g in small intestines and systemic organs in *Stat1* KO 129sv mice was about 10-fold higher than in wild-type 129sv mice, but the replication of rD6/2-2g-NSP1-null in these tissues in wild-type 129sv mice was similar to that of *Stat1* KO 129sv mice (Fig. 5 and 8). These data suggest that NSP1 protein contributes to virus replication but that this contribution appears to be independent of IFN signaling during RV infection *in vivo*. We also found that the replication levels of the heterologous rSA11-NSP1-null and rSA11 simian RVs were comparable in wild-type 129sv mice, but the replication of rSA11 was increased in *Stat1* KO 129sv mice (Fig. S5). Unlike rD6/2-2g, we did not see a recovery of rSA11-NSP1-null in wild-type mice, probably because the replication level was too low to permit generation and selection of sufficient mutations by the viral polymerase.

Although this study identifies an essential role of the NSP1 protein in promoting RV replication *in vivo*, the precise mechanisms of this promoting phenotype remain to be further explored. Our data suggest that NSP1 likely facilitates virus replication independently of the IFN and inflammasome signaling and beyond one replication cycle. Interestingly, NSP1 has been shown to be localized to the nucleus during viral infection, and this relocalization disrupted the function of promyelocytic nuclear bodies, which was related to host stress responses (29). More recently, NSP1 has been linked to the inhibition of the transcription (30) but not the translation (14) of IRF1. It will be of interest to determine whether the NSP1-IRF1 signaling pathway is involved in RV replication *in vivo*. In several ways, RV NSP1 resembles influenza virus NS1, another multifunctional protein that potentially targets protein kinase R (31) and autophagy (32). In this sense, several other arms of the innate immune signaling remain to be examined. Meanwhile, it is also plausible that NSP1 contributes to RV infection in ways other than the innate immune antagonism. For instance, NSP1 associates with viral RNA (33) and may facilitate viral assembly or egress beyond the replication step.

In future studies, it will also be interesting to investigate the exact domains of NSP1 responsible for its proviral replication functions *in vivo*. In light of the perplexing finding that the replication of NSP1-null RV is not rescued in *Stat1* KO mice, deletion of the IRF3 binding site in murine RV NSP1 or swapping that with the β-TrCP recognition motif could be revealing. In addition, since NSP1 is involved in RV host range restriction, one can study the NSP1 functionality in the context of a homologous virus infection by replacing the murine RV NSP1 with NSP1s derived from heterologous RV strains. With an optimized RG system and fully virulent murine RVs, we expect to uncover the physiological functions of other RV-encoded viral factors. Finally, while our studies here show that NSP1 is pivotal for rotavirus replication *in vivo* and represents a logical viral target for replication restriction-based attenuation, site-directed mutagenesis may not be the optimal approach to disrupt NSP1 expression due to the quick and complete reversion. Moving forward, we plan to generate recombinant RVs that lack the complete or a portion of the NSP1 coding region to prevent the reversion to virulent parental strains. We will be able to test the replication, pathogenesis, and immunogenicity of the new NSP1-truncated RVs as novel vaccine candidates against subsequent RV infections in small animal models. We anticipate that a deeper understanding of the RV-host interactions and RV pathogenesis *in vivo* will guide the development of novel next-generation RV vaccine candidates with improved efficacy in developing countries and higher compatibility with an immunocompromised population.

## MATERIALS AND METHODS

### Cells and viruses.

The rhesus monkey kidney epithelial MA104 cells were grown in medium 199 (Gibco) supplemented with 10% heat-inactivated fetal bovine serum (FBS) (VWR), 100 U/ml penicillin, 100 μg/ml streptomycin, and 0.292 mg/ml l-glutamine. The BHK-T7 cell line, a baby hamster kidney cell line stably expressing T7 RNA polymerase, was kindly gifted by Ursula Buchholz (Laboratory of Infectious Diseases, NIAID, NIH, USA) and was cultured in Dulbecco’s modified Eagle’s medium (DMEM) (Gibco) supplemented with 10% heat-inactivated FBS, 100 U/ml penicillin, 100 μg/ml streptomycin, and 0.292 mg/ml l-glutamine, and also 0.3 mg/ml G418 (Promega) was added to the culture medium at every other passage. MA104 cells stably expressing parainfluenza virus 5 V protein and bovine viral diarrhea virus N protease were cultured in medium 199 supplemented with 10% heat-inactivated FBS, 100 U/ml penicillin, 100 μg/ml streptomycin, 0.292 mg/ml l-glutamine. Blasticidin (10 μg/ml) and puromycin (10 μg/ml) were added to the medium at every other passage as described previously (22).

The simian RV SA11 strain and the murine RV D6/2 strain were propagated as described previously (7, 19). Briefly, RV stock was activated with 5 μg/ml trypsin (Gibco Life Technologies, Carlsbad, CA) for 20 min at 37°C. The activated RVs were incubated with MA104 cells, which were washed twice with serum-free medium 199 for 1 h at 37°C. Then, the viruses were removed and the new serum-free medium 199 supplemented with 0.5 μg/ml trypsin was added to the MA104 cells. Virus titers were determined by a standard plaque assay in MA104 cells.

### Sequencing of the murine RV D6/2 strain.

Viral particle enrichment of cell culture supernatant was performed based on the NetoVIR protocol (34). Briefly, cell culture supernatant was centrifuged for 3 min at 17,000 × *g* and filtered with a 0.8-μm polyethersulfone (PES) filter (Sartorius). The filtrate was treated with Benzonase (Novagen) and micrococcal nuclease (New England BioLabs) at 37°C for 2 h to remove the free-floating nucleic acids. Subsequently, samples were extracted using the QIAamp viral RNA minikit (Qiagen) according to the manufacturer's instructions, without addition of carrier RNA to the lysis buffer. Reverse transcription and second-strand synthesis were performed by an adjusted version of the whole-transcriptome amplification (WTA2) protocol (Sigma-Aldrich), as described previously (35). A sequencing library was constructed with the Nextera XT library preparation kit (Illumina). The size of the library was checked with Bioanalyzer (Agilent Technologies) with a high-sensitivity DNA chip, and the 2 nM pooled libraries were sequenced on an Illumina NextSeq 500 platform (2 × 150-bp paired ends).

Low-quality reads, ambiguous bases, and primer and adaptor sequences were removed from the paired-end reads with Trimmomatic with default parameters (36). Trimmed reads were *de novo* assembled with metaSPAdes from SPAdes software using 21, 33, 55, and 77 k-mer lengths (37). The obtained contigs were annotated with DIAMOND against a nonredundant protein database (38). Contigs annotated as “rotavirus” were extracted. The obtained sequences were verified *in silico* by remapping the trimmed reads to the obtained contigs using BWA software (39).

### Construction of a T7 plasmid carrying mutant gene 5 of D6/2 or SA11.

To rescue an NSP1-deficient murine-like RV, we generated a pT7-D6/2-NSP1-null virus via the QuikChange II site-directed mutagenesis kit (Agilent Technology) based on pT7-D6/2-NSP1 (22). Briefly, the AAG and TGC codons in the NSP1 open reading frame (ORF) of D6/2 were replaced with stop codons TAG and TGA at nucleotide position 43 to 45 and 52 to 55 in pT7-D6/2-NSP1. The mutant primers used were rD6/2-2g-NSP1-null forward primer, 5′-GTGTTAGCCATGGCGGCCTTTTAGGACGCGTGATTCCATTACAGAAGG-3′, and rD6/2-2g-NSP1-null reverse primer, 5′-CCTTCTGTAATGGAATCACGCGTCCTAAAAGGCCGCCATGGCTAACAC-3′. To rescue the NSP1-deficient simian RV SA11, we generated the plasmid pT7-SA11-NSP1-null using the same strategies described above. The mutant primers used were SA11-NSP1-null forward primer, 5′-GCTACTTTTAAAGATGCATGCTTTTAATAGCGTAGATTAACTGCTTTAAATCGG-3′, and SA11-NSP1-null reverse primer, 5′-CCGATTTAAAGCAGTTAATCTACGCTATTAAAAGCATGCATCTTTAAAAGTAGC-3′.

### Generation of recombinant murine-virus-like and simian RVs.

Recombinant rD6/2-2g and rD6/2-2g-NSP1-null were generated according to the optimized entirely plasmid-based RG system described recently (22). Briefly, 0.4 μg of pT7-SA11-VP1, pT7-D6/2-VP2, pT7-D6/2-VP3, pT7-D6/2-VP4, pT7-D6/2-VP6, pT7-D6/2-VP7, pT7-D6/2-NSP1 (or pT7-rD6/2-NSP1-null), pT7-D6/2-NSP3, and pT7-SA11-NSP4; 1.2 μg of pT7-D6/2-NSP2 and pT7-D6/2-NSP5; 0.8 μg of the helper plasmid C3P3-G1; and 14 μl TransIT-LT1 (Mirus) transfection reagent were mixed together and transfected into BHK-T7 cells in a 12-well plate. Eighteen hours later, the transfected BHK-T7 cells were washed twice with FBS-free DMEM and then supplemented with 800 μl fresh FBS-free DMEM, and 24 h later, 1 × 10^5^ MA104 N*V cells in 200 μl FBS-free DMEM along with 0.5 μg/ml trypsin were added to the transfected BHK-T7 cells for another 3 days. After that, mixed cells were frozen and thawed 3 times. The rescued virus was propagated for two passages in MA104 cells in a 6-well plate, and then the virus was propagated in a T75 flask to produce the virus stock.

The recombinant SA11 and SA11-NSP1-null were rescued using the same protocol described above, expect that the plasmids used were pT7-SA11-VP1, pT7-SA11-VP2, pT7-SA11-VP3, pT7-SA11-VP4, pT7-SA11-VP6, pT7-SA11-VP7, pT7-SA11-NSP1 (or pT7-SA11-NSP1-null), pT7-SA11-NSP2, pT7-SA11-NSP3, pT7-SA11-NSP4, and pT-SA11-NSP5.

### Purification of RV particles by sucrose gradient centrifugation.

RVs were concentrated by sucrose cushion as described previously (40). Briefly, RVs propagated in T75 flasks were harvested by repeating the freeze-thaw cycle three times. Then, we clarified the crude lysate of cell debris by centrifugation at 3,000 × *g* for 1 h at 4°C. After that, 8 ml of clarified RVs was placed into a 10-ml SW44 ultracentrifuge tube, and 2 ml 40% (wt/vol) sucrose was carefully added to the bottom of the ultracentrifuge tube and subjected to centrifugation at 35,000 rpm for 3 h at 4°C. At last, we removed the supernatant and added 200 μl FBS-free medium 199 to resuspend the concentrated RVs at 4°C overnight.

### Electrophoretic analysis of viral genomic dsRNAs.

Viral genomic dsRNAs were extracted from sucrose cushion-concentrated viruses using TRIzol reagent (Thermo Scientific) according to the manufacturer’s protocol (41). Then, the dsRNAs were mixed with gel loading dye, purple (6×) (New England Biolabs). Samples were loaded into a 4 to 15% precast polyacrylamide gel and run for 3 h at 180 V. The gel was stained for 1 h with 0.1 μg/ml ethidium bromide and visualized by the ChemiDoc MP imaging system (Bio-Rad).

### Restriction enzyme digestion and sequencing analysis.

The total RNA of the recombinant rD6/2-2g and rD6/2-2g-NSP1-null virus stocks and stool samples was extracted by TRIzol. Total RNA was reverse transcribed to cDNA using a high-capacity cDNA reverse transcription kit with RNase inhibitor (Applied Biosystems) according to the user guide. Briefly, 0.8 μg of RNA, 2 μl of 10× reverse transcription (RT) buffer, 0.8 μl of 100 mM deoxynucleoside triphosphate (dNTP) mix, 2 μl of RT random primers, 0.1 μl of RNase inhibitor, 0.1 μl of MultiScribe reverse transcriptase, and a flexible amount of nuclease-free H_2_O were added to the 20-μl reaction mixture. The reverse transcription thermocycling program was set at 25°C for 10 min, 37°C for 2 h, and 85°C for 5 min.

NSP1 5′-end fragments were amplified by Phusion Hot Start II DNA polymerase (Thermo Scientific) following the manufacturer’s guide. The primers used for PCR were NSP1 forward primer, 5′-GTCTTGTGTTAGCCATGGC-3′, and NSP1 reverse primer, 5′-CAGCGGTTAAAGTGATCGG-3′. PCR products were gel purified using the QIAquick gel extraction kit (Qiagen). NSP1 fragments of 0.1 μg were digested by restriction enzyme HinfI (New England Biolabs [NEB]) for 1 h at 37°C. The enzyme-digested products were separated by 2% agarose gel electrophoresis, stained by ethidium bromide, and visualized by the ChemiDoc MP imaging system (Bio-Rad). A separate set of purified NSP1 fragments were sent for Sanger sequencing.

### Immunoblotting.

MA104 cells in a 24-well plate were infected by rD6/2-2g or rD6/2-2g-NSP1-null at an MOI of 3 for 6 h. Then, uninfected and infected MA104 cells were washed twice with ice-cold phosphate-buffered saline (PBS; Thermo Scientific) and lysed in RIPA buffer (150 mM NaCl, 1.0% IGEPAL CA-630, 0.5% sodium deoxycholate, 0.1% SDS, 50 mM Tris, pH 8.0; Sigma-Aldrich) supplemented with 1× protease inhibitor cocktail (Thermo Scientific) for 30 min at 4°C. After that, cell debris was removed by centrifugation at 12,000 × *g* for 10 min at 4°C. Samples were resolved in precast SDS-PAGE gels (4 to 15%; Bio-Rad) and transferred to a nitrocellulose membrane (0.45 μm; Bio-Rad). The membrane was incubated with blocking buffer (5% bovine serum albumin [BSA] diluted in PBS supplemented with 0.1% Tween 20) for 1 h at room temperature. Then, the membrane was incubated with anti-IRF3 rabbit monoclonal antibody (CST; catalog no. 4302; 1:1,000), anti-RV VP6 mouse monoclonal antibody (Santa Cruz Biotechnology; sc-101363; 1:1,000), and anti-glyceraldehyde-3-phosphate dehydrogenase (GAPDH) rabbit monoclonal antibody (CST; catalog no. 2118; 1:1,000), followed by incubation with anti-mouse IgG (CST; catalog no. 7076; 1:5,000) or anti-rabbit IgG (CST; catalog no. 7074; 1:5,000) horseradish peroxidase (HRP)-linked antibodies. The antigen-antibody complex was detected using Clarity Western ECL substrate (Bio-Rad) and the ChemiDoc MP imaging system according to the manufacturer’s manuals.

### RT-qPCR.

RT-qPCR was performed using the above cDNA as described previously (23). The expression level of housekeeping gene GAPDH was quantified by 2× SYBR green master mix (Applied Biosystems), and NSP5 was measured by 2× TaqMan Fast Advanced master mix (Applied Biosystems). The primers used in this study were as follows: human GAPDH forward primer, 5′-GGAGCGAGATCCCTCCAAAAT-3′, and reverse primer, 5′-GGCTGTTGTCATACTTCTCATGG-3′; mouse GAPDH forward primer, 5′-TCTGGAAAGCTGTGCCGTG-3′, and reverse primer, 5′-CCAGTGAGCTTCCCGTTCAG-3′; and NSP5 forward primer, 5′-CTGCTTCAAACGATCCACTCAC-3′, reverse primer, 5′-TGAATCCATAGACACGCC-3′, and probe, 5′-CY5/TCAAATGCAGTTAAGACAAATGCAGACGCT/IABRQSP-3′. For the viral load measurement in the small intestinal segments and systemic organs, GAPDH levels were quantified and used to normalize the different cell numbers in the tissues harvested. The *y* axis stands for the percentage of NSP5 mRNA levels relative to GAPDH levels, plotted on a log_10_ scale.

### Plaque assay.

The plaque assay was performed as described previously (42). Briefly, 1 × 10^5^ MA104 cells/ml were seeded in a 6-well plate, and virus samples were serially diluted 10-fold and incubated with the confluent MA104 cells for 1 h at 37°C. Then, samples were replaced by FBS-free medium 199 with 0.1% agarose supplemented with 0.5 μg/ml trypsin and put back to 37°C. Plaques were visualized at day 3 to day 5 postinoculation by 0.0165% neutral red staining. In order to measure the size of the plaques, we recorded more than 75 plaques with the microscope (Echo) in two different experiments. Then, the diameters of the plaques were calculated with the annotation tool of the microscope.

### Focus-forming unit assay.

The focus-forming assay was conducted as described previously (41). Briefly, 1 × 10^5^ MA104 cells/ml were seeded in a 96-well plate, and then virus samples were serially diluted 5- or 10-fold and incubated with a monolayer of MA104 cells for 10 h or overnight at 37°C. Then, cells were fixed with 10% formalin, followed by permeabilization with 1% Tween 20. After that, cells were incubated with anti-rotavirus capsid mouse monoclonal antibody and anti-mouse HRP-linked antibodies. The foci were stained by 3-amino-9-ethylcarbazole HRP substrate (Vector Laboratories) and stopped by washing twice with PBS.

### Virus competition assay.

The virus competition assay was conducted as described previously (43). Briefly, fully confluent MA104 cells were infected by rD6/2-2g and rD6/2-2g-NSP1-null at an MOI of 0.01 or 3 for 1, 8, and 24 h at 37°C. The total RNA was extracted from the infected MA104 cells, and the 5′-end fragments of NSP1 were amplified as described above. The amplified fragments were gel extracted and analyzed by Sanger sequencing.

### Mouse infection.

Wild-type 129S1/SvImJ, *Stat1* KO, wild-type C57BL/6, and *Asc* KO mice were purchased from the Jackson Laboratory and Taconic Biosciences and bred locally at the Washington University in St. Louis (WUSTL) BJC Institute of Health (BJCIH) vivarium. Five-day-old suckling pups were orally infected with rescued rD6/2-2g (1.5 × 10^3^ FFU) and rD6/2-2g-NSP1-null (1.5 × 10^3^ FFU) (44). Diarrhea was evaluated from day 1 to day 12 postinfection. In the meantime, feces from infected mice were also collected, and the focus-forming assay was used to titrate RV in stool samples. Briefly, 50 μl PBS with calcium and magnesium was added to the 1.5-ml Eppendorf tubes, and the weight was recorded. After we collected the feces, these tubes were weighed again, and the stool samples were homogenized before we made the serial dilution to conduct the focus-forming assay. Duodenum, jejunum, ileum, blood, mesenteric lymph node, and liver were collected from inoculated pups at day 2 postinfection, immediately placed in liquid nitrogen, and stored at −80°C until use. RNA was extracted from those tissues using the RNeasy Plus minikit (Qiagen) according to the manufacturer’s protocol. RT-qPCR was used to measure RV NSP5 expression levels, and FFU assays were used to quantify the amount of infectious virus in the stool samples as described previously (45).

### Statistical analysis.

Bar graphs in Fig. 2A to C and Fig. 3A and B were displayed as means ± standard deviations (SD). Bar graphs in Fig. 4B, Fig. 5A to E, Fig. 6A to C, Fig. 7B, Fig. 8A to E, and Fig. S2, S4, and S5B and C in the supplemental material were displayed as means ± standard errors of means (SEM). Statistical significance in Fig. 2A to C, Fig. 3A and B, Fig. 5B to E, Fig. 8B to E, and Fig. S5C was analyzed by unpaired Student’s *t* test using GraphPad Prism 9.1.1. Statistical significance in Fig. 4B, Fig. 7B, and Fig. S2 and S5B was calculated by two-way analysis of variance (ANOVA). Statistical significance in Fig. 5A, Fig. 6A to C, Fig. 8A, and Fig. S4A and B was analyzed by Mann-Whitney U test (*, *P* < 0.05; **, *P* < 0.01; ***, *P* < 0.001; n.s., not significant).
