# Supplementary figures and images for: Rotavirus NSP1 Contributes to Intestinal Viral Replication, Pathogenesis, and Transmission
Source: mBio. 2021 Dec 14;12(6):e03208-21. doi: 10.1128/mBio.03208-21 (PMC8669464; doi:10.1128/mBio.03208-21)

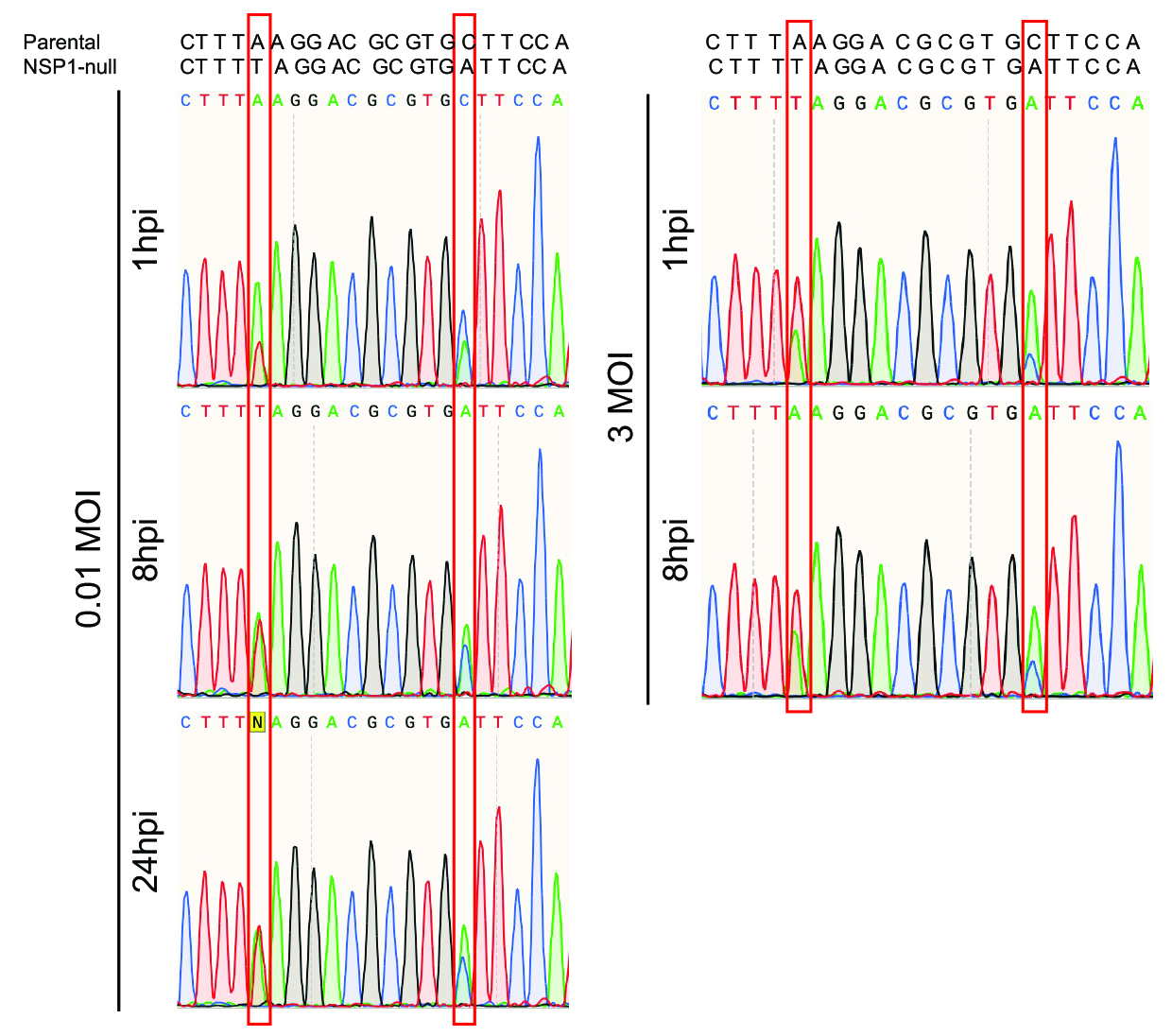

Supplement: FIG S1 [file mbio.03208-21-sf001.tif]

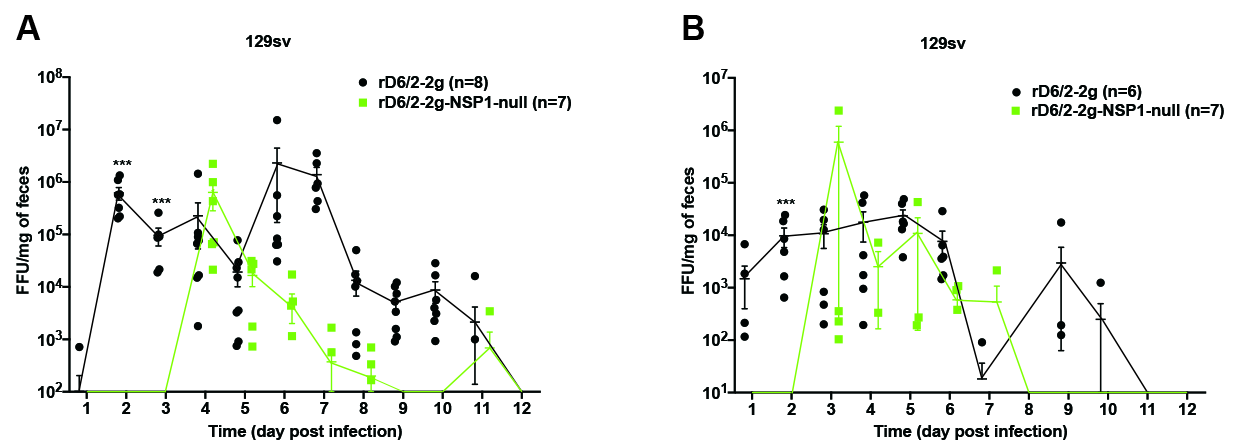

Supplement: FIG S2 [file mbio.03208-21-sf002.tif]

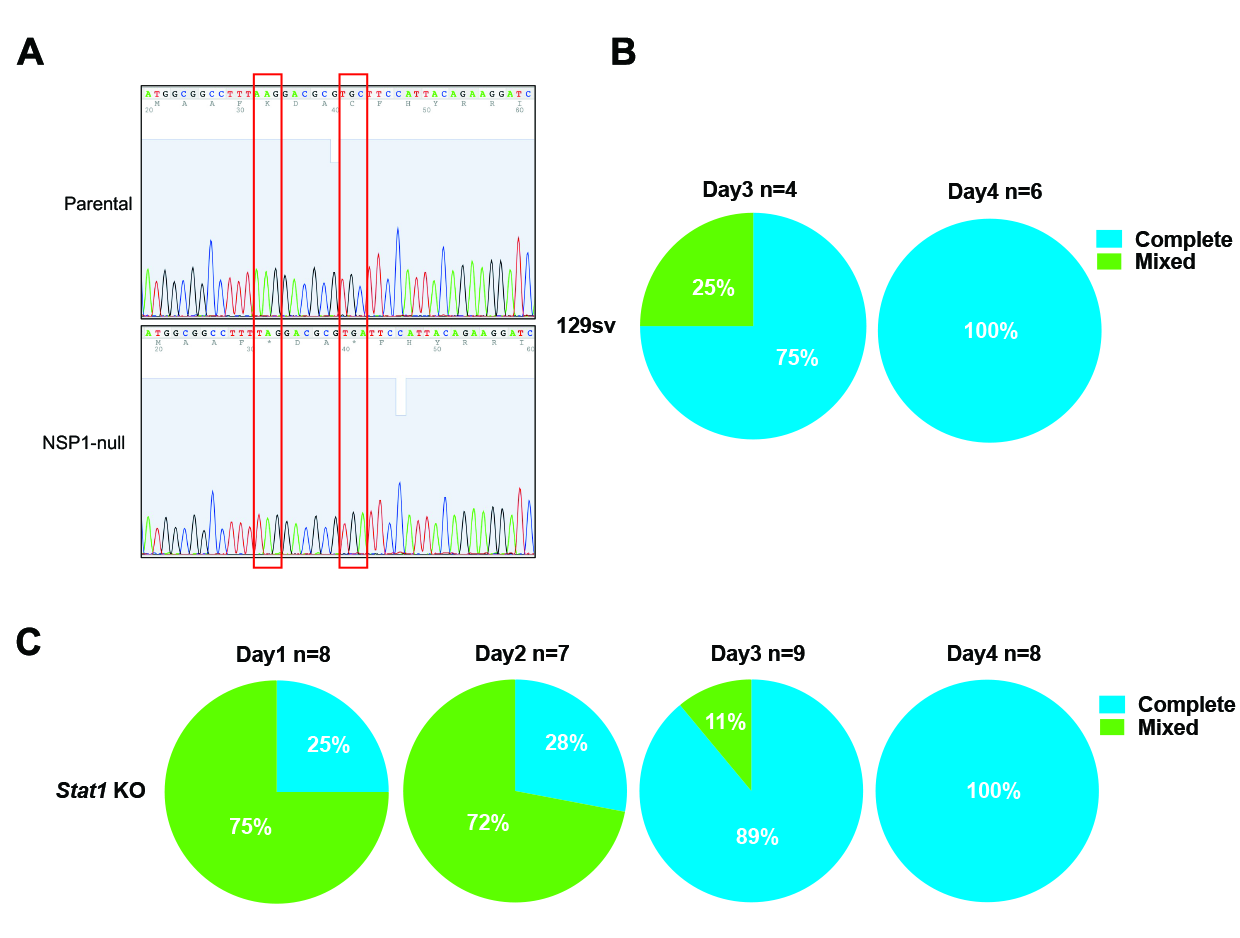

Supplement: FIG S3 [file mbio.03208-21-sf003.tif]

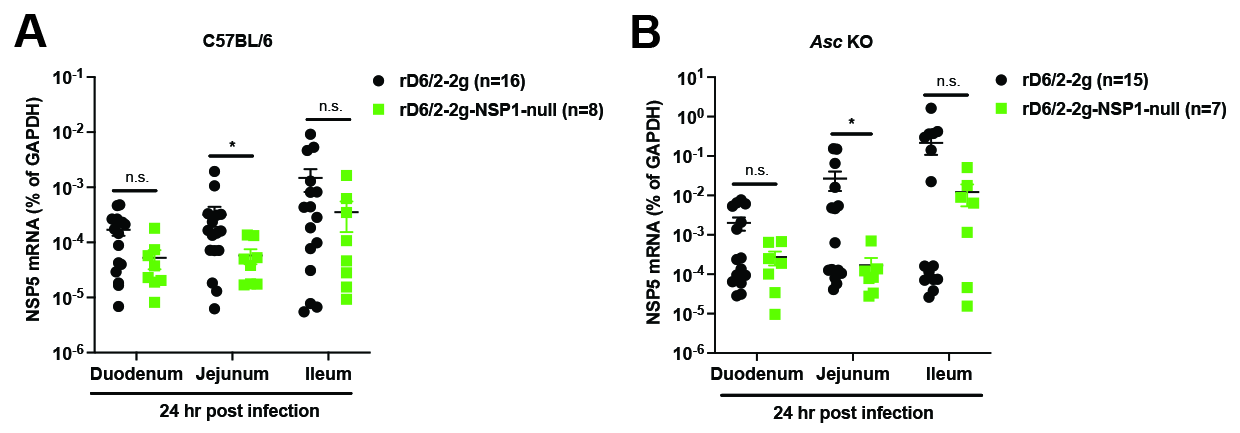

Supplement: FIG S4 [file mbio.03208-21-sf004.tif]

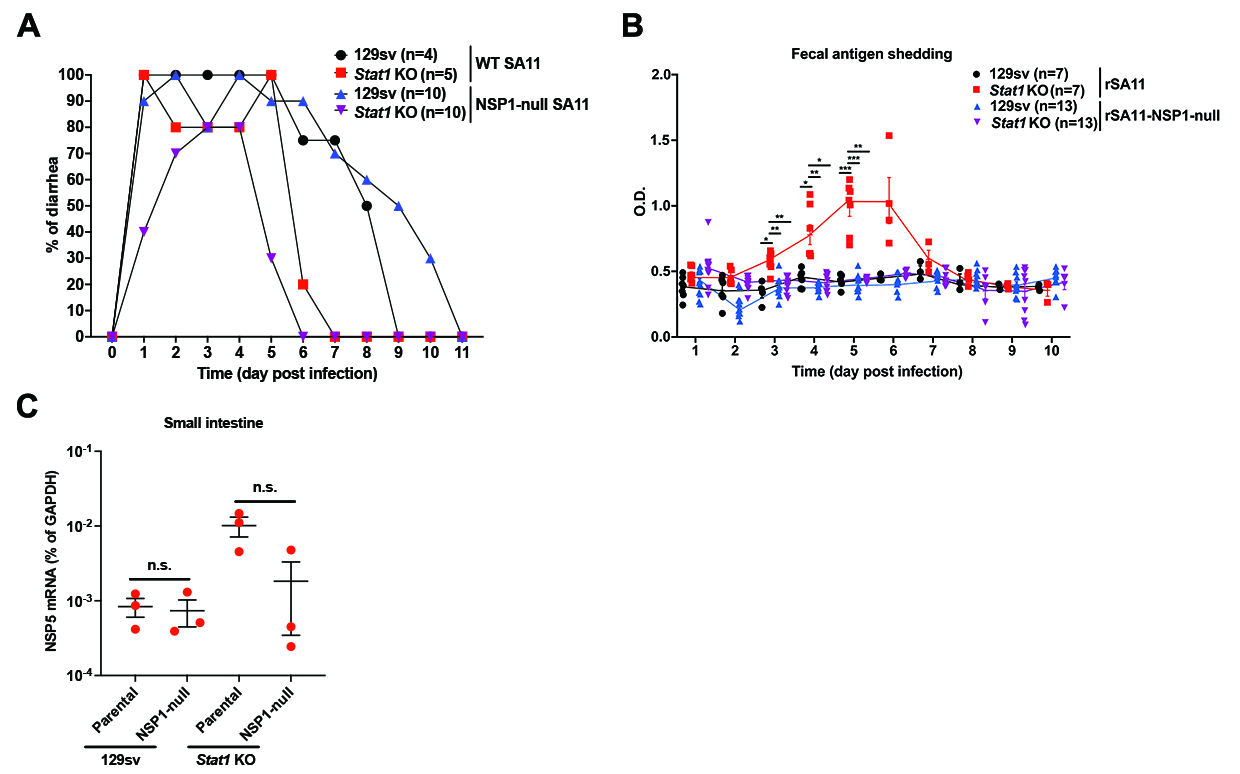

Supplement: FIG S5 [file mbio.03208-21-sf005.tif]
